# Revisiting the biophysical aspects of extracellular-matrix-mimicking hydrogels: what cells see *vs.* what cells feel

**DOI:** 10.1039/d5bm00210a

**Published:** 2025-08-22

**Authors:** Jingchao Sui, Sarah Pragnere, Nicholas A. Kurniawan

**Affiliations:** a Department of Biomedical Engineering, Eindhoven University of Technology PO Box 513 5600 MB Eindhoven the Netherlands kurniawan@tue.nl; b Institute for Complex Molecular Systems, Eindhoven University of Technology PO Box 513 5600 MB Eindhoven the Netherlands

## Abstract

The extracellular matrix (ECM) is critical in regulating cell behavior and tissue function. This recognition has driven the development of ECM surrogates to better understand cell–ECM interactions and advance biomedical applications. Hydrogels are promising candidates for this purpose due to their biocompatibility, tunability, and ability to embed cells in 3D environments. While early efforts in the design of ECM-mimicking hydrogels focused on macromolecular type, it is now clear that their biophysical parameters, such as polymer molecular weight, fibrous *versus* non-fibrous structures, pore size, and mechanical properties, significantly influence cell behavior. Understanding the interplay of these factors is crucial for the rational design of biomaterials, but remains challenging given the complexity of hydrogel systems and the rapid pace of new findings. This review critically evaluates hydrogels as ECM mimics for 3D cell cultures. We revisit key ECM properties to replicate, examine how hydrogel design can meet these needs, and summarize the impact of biochemical, structural, and mechanical features on cell behavior. We also explore how structural and mechanical properties—what cells “see” and “feel”—are interrelated and jointly affect cell function. Our analysis concludes that strategic combinations of polymeric materials will play an important role for next-generation hydrogels to replicate physiological conditions and independently enable precise control over key parameters. These advancements will enhance our understanding of cell–ECM interactions and support the development of innovative biomaterials for tissue engineering and regenerative medicine.

## Abstract 2

This review revisits key properties of the extracellular matrix to be replicated in hydrogels for 3D cell culture and summarizes the interplay between hydrogel structural and mechanical properties, as well as their combined impact on cell function.

## Graphical abstract

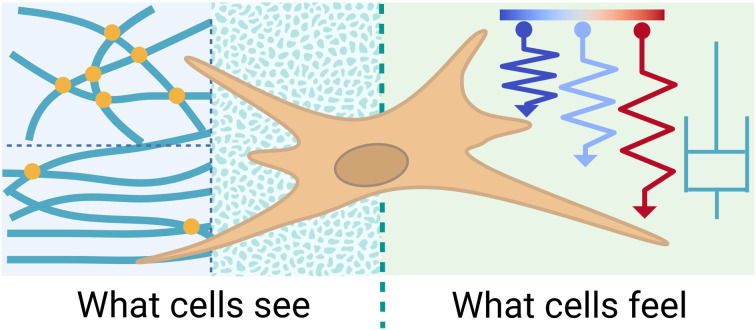

## 1. Introduction

The tissue environment in which cells reside is composed of extracellular matrix (ECM). By now, it is well established that the ECM influences cell behavior and tissue function as much as the intrinsic cellular properties. Specifically, the ECM acts as an active regulatory milieu for directing cell functions, such as growth, differentiation, and cell–cell communication.^1–3^ Moreover, the ECM forms one of the three key pillars in tissue engineering and regenerative medicine, alongside cells and regulatory signals.^4^ Therefore, the need for an ECM surrogate or equivalent is recognized, both for gaining a fundamental understanding of cell behavior in tissue environments and for developing biomedical solutions and applications targeting ECM-mediated cell and tissue functions. Hydrogels are promising as they are biocompatible and tunable, allowing cell embedding in physiologically relevant 3D environments.

Historically, the first system of hydrogels approaching a 3D cell culture appeared in the 1970s and was based on floating collagen gels,^5–8^ followed soon after by systems based on reconstituted basement membranes (currently known as Matrigel).^8,9^ Around the 1990s, hydrogels evolved into a more complex system incorporating non-animal-origin biopolymers such as alginate^10,11^ for biocompatibility, ease of crosslinking, mild gelation conditions suitable for embedded cells, and their relatively low cost. Synthetic polymers such as dimethylaminoethyl methacrylate (DMAEMA) and methacrylic acid (MAA)^12^ were then incorporated for better controllability and superior mechanical properties compared to collagen. Hybrid hydrogels^13^ surfaced as attempts to combine the controllability and advantages of both natural proteins and synthetic components.

From a broader perspective, the use of hydrogels as a biomaterial to embed cells has ushered in the era of three-dimensional (3D) cell culture. Indeed, unlike two-dimensional (2D) culture systems or scaffolds, embedding cells in hydrogels provide a 3D mechanical environment to the cells, which better mimics the *in vivo* 3D environment.^14^ Pioneering studies and observations highlighted the impact of dimensionality on cell behavior. Firstly, 2D culture places cells in a planar environment, which imposes restrictions on cell migration along the direction perpendicular to the plane. Besides, cells cultured on 2D plans lack direct exposure to physical confinement as well as the spatial gradients of soluble factors from the surrounding microenvironment, which is present in the 3D environment.^15^ Secondly, (ventral/dorsal) polarization of cells such as mesenchymal cells in 2D cultures does not appear when these cells are embedded in 3D hydrogels.^16^ All these aspects influence cellular processes such as cell phenotypic fate. For example, mouse and human mammary epithelial cells embedded in hydrogels maintained a normal phenotype *in vitro*, while these cells display tumor-like characteristics when cultured in a traditional 2D environment.^6,17,18^ Later studies have shown that dimensionality also impacted the *in vitro* maintenance of the phenotype of fully differentiated cells, such as chondrocytes^19^ or osteocytes.^20^ These findings thus indicate the importance of 3D culture of cells embedded in physiologically relevant ECM-mimicking hydrogels for better recapitulating *in vivo* cellular environments and preserving cell native phenotypes.

Initially, hydrogel development for cell culture focused on selecting the macromolecular type. However, the increasing complexity of hydrogel-based systems has revealed that cell responses are also strongly influenced by other, more biophysically oriented parameters, such as polymer molecular weight, fibrous *versus* non-fibrous structures, pore dimensions, and mechanical properties. The role of each of these parameters, especially in combinatorial permutation with each other, in directing cell behavior and the eventual tissue function is far from understood, even though this knowledge is crucial for the rational design of biomaterials. Moreover, the rapid pace at which various observations of cell response in different types of hydrogel environments are reported has often made it difficult not only to identify which parameters should be considered but also to distill the key determining parameters.

In this review, we will therefore take a step back and critically examine the use of hydrogels as an ECM mimic for 3D cell cultures. We will start by revisiting the properties of the ECM to be reproduced and how this need can be met with the appropriate hydrogel design. Then we will discuss how the structural and mechanical properties of the hydrogels (*i.e.*, what cells see and what cells feel, respectively) are interrelated and strongly impact cell function.

## 2. What do we want to reproduce? key properties of the ECM

**Fig. 1:**
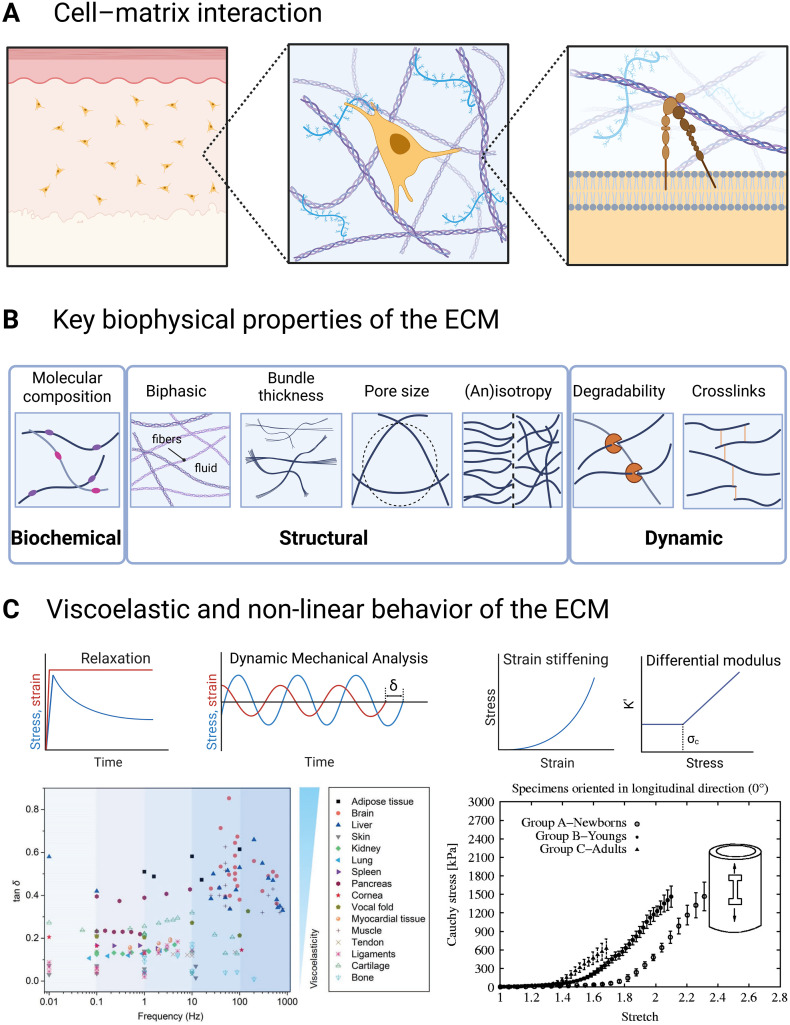
Key properties of the ECM. (A) Tissues (left) are composed of cells embedded in ECM (middle). Cells can bind to ECM components such as fibrous collagen I, fibronectin, and hyaluronic acid chain *via* specific receptors such as integrins (right). (B) Key biochemical (molecular composition), structural (biphasic material, bundle thickness, pore size, and (an)isotropy), and dynamic (degradability and degree of crosslinks) parameters of the ECM that influence the mechanical properties and cell behavior. (C) Example of the unique mechanical behaviors of biological tissues. Left: viscoelastic behavior can be measured by stress-relaxation or dynamic mechanical analysis experiments. In native tissues, viscoelasticity (represented by the values of tan *δ*) varies as a function of frequency (reproduced from ref. 21 with permission from WILEY, copyright 2021). Lower values of tan *δ* reflect more elastic behavior while higher values reflect increased viscous behavior. Right: nonlinear stress–strain behavior can be visualized by plotting the stress–strain curves, or by computing the differential modulus *K*′ as a function of stress (or strain). The nonlinear behavior is illustrated with stress–strain curves of human thoracic descending aortas (reproduced from ref. 22 with permission from Taylor & Francis Group, copyright 2012).

The ECM plays a double role in cell and tissue physiology. At the cell scale, the ECM acts as a physical support and dynamic environment for cells to attach to and navigate in. At the tissue scale, the ECM provides mechanical strength, structure, and resilience needed for bodily functions, but which individual cells lack. To enable this dual role and simultaneously meet the diverse tissue-specific physiological demands, Nature has finetuned the ECM from an ingredient set of macromolecules with unique properties, built with unique composition and physicomechanical properties (Fig. 1A). To establish the specifications that 3D ECM-mimicking hydrogel models must meet, it is necessary first to understand the key properties of the native ECM. We categorize them into three main groups: biochemical, structural, and mechanical properties.

### 2.1. Biochemical composition

The ECM is composed of proteins, mainly fibrous, in a ground substance. The most abundant proteins are collagens, which constitute approximately 30% of the total ECM in the human body, particularly collagen types I, II, and III.^2^ The mechanical function of collagen is mainly to provide resistance to tension. For example, in arteries undergoing high blood pressure and in ligaments of moving joints, collagen helps to transmit forces and prevent excessive stretching.^23^ Other major ECM proteins include elastin and fibronectin, and in the case of wound healing, fibrin. Elastin is crucial in tissues that are physiologically required to undergo elastic deformation, such as lungs, skin, and arteries,^23^ and makes up about 50% of dry weight in arteries.^24^ Fibronectin is a glycoprotein that primarily functions as a connector for cell–ECM, ECM–ECM, and ECM–growth factor. Therefore, although fibronectin has a negligible direct role in the mechanical properties of the ECM, it plays a major role in cell binding to the ECM and the transmission of mechanical forces from the cytoskeleton to the ECM *via* integrins.^25^ Laminin is another protein that plays a crucial role in connective tissues, particularly in the basement membrane where it acts as a barrier between endothelial or epithelial cells and connective tissues.^26^ It also binds to other ECM components and cells, forming a supramolecular network.^2^ The ground substance is composed of proteoglycans, large molecules comprised of a core protein to which glycosaminoglycans (GAGs) are bound. Due to their negative charges, GAGs bind to water. For example, one molecule of hyaluronic acid (HA), a major GAG component, can bind up to 500 molecules of water, which contributes to lubrication and resistance to compression, a critical function in joints.^2,23^

ECM components such as fibronectin and GAGs can bind to various other molecules, such as growth factors, and are involved in the activation of several biological processes.^27^ For example, heparan sulfate, a type of GAG, can bind to vascular endothelial growth factor and fibroblast growth factor-2 (FGF-2), and its binding to FGF-2 is required for the corresponding signaling and cellular response.^28^ ECM can directly bind and release growth factors, thereby concentrating them in the vicinity of cells; it can also sequester growth factors to protect them from degradation.^28,29^ ECM can regulate (enhance^30^ or inhibit^31^) the activity and signaling of growth factors, and reciprocally, growth factors regulate the ECM by stimulating cells synthesis or degrading ECM components.^28^ Extensive overview of ECM–growth factor interactions can be found in review articles by Taipale and Keski-Oja^32^ and by Schultz and Wysocki;^28^ specifically for fibronectin–growth factor interactions, see review articles by Clark and colleagues.^30,33^

As mentioned above, specific components of the ECM enable cell adhesion. Cells bind to ECM components *via* specific amino acid sequences known as cell-adhesion peptides. Different adhesion peptides activate different receptors on the cell membrane, which in turn trigger different cellular responses (Table 1). Notably, integrins, key transmembrane receptors found only in animal cells, connect the ECM to the inside of the cell, and integrin activation facilitates different bidirectional signal transmission pathways: from the outside toward the inside of cells (outside-in), and from the inside of cells toward the outside (inside-out).^23^ The binding of cells to ECM *via* integrin also enables cells to respond to growth factors, and to increase the synthesis of growth factors.^28^

**Table 1:** Examples of common extracellular matrix components, their adhesion peptides, and the cellular receptor they bind to

Matrix component	Binding sequence	Associated cell membrane receptors	Examples of effects of receptor activation
Collagen I	GFOGER	α_1_β_1_, α_2_β_1_, α_3_β_1_, α_10_β_1_, α_11_β_1_, discoidin domain receptors (DDR)^2^	β1 subunits are essential for tissue repair.^34^ α_11_β_1_ is a major collagen receptor on fibroblastic cells^2^ while α_10_β_1_ binds only to collagen II
			DDRs regulate migration, proliferation, differentiation, survival, and MMP expression
Fibronectin	GRGDS	α_ν_β_3_, α_ν_β_5_	α_ν_β_3_ activates cell contraction^35^ and fibroblast activation^36^
Fibrin	RGD	α_ν_β_3_, α_3_β_2_^26,37^	
Laminin	IKVAV	α_3_β_1_, α_6_β_1_, α_6_β_4_, α_6_β_7_, α_7_β_1_, α_1_β_1_, α_2_β_1_, α_10_β_1_^2^	Integrins α_3_β_1_ and α_6_β_4_ can either suppress or promote tumor development and progression^38^
GAGs	—	CD44, RHAMM/CD168, ICAM-1^2^	The binding of hyaluronic acid (HA) to CD44 and RHAMM guides muscle development,^2^ and chondroitin sulfate modulates axon growth^27^

Although the main macromolecular components of the ECM are the same throughout the human body, the ECM is highly tissue-specific. For example, the bone matrix is dense and stiff, whereas the ECM in soft connective tissue forms a loose mesh. This diversity and tissue specificity of ECM arise from variations in its specific composition ratios and structural morphology. Therefore, in the next section, we will briefly discuss the main morphological features and parameters of interest in the ECM.

### 2.2. Structure

Fibers play a critical role in the ECM structure. The prevalence of fibrous structures is largely attributed to the high concentration of fibrous proteins in the ECM. Mechanically, a relaxed network of elastin and collagen in the ECM accommodates a wide range of tensile stresses through its hierarchical structure, which enables fiber bending, stretching, and alignment.

Collagen fibers exhibit a highly hierarchical structure. Three polypeptide chains wrap together into tropocollagen, a triple-helix line approximately 300 nm in length and 1.2–1.5 nm in diameter. These tropocollagens assemble to form fibrils, which in turn assemble into fibers, with a diameter of about 10 μm and a length of millimeters. This hierarchical fibrillar structure allows for mechanical adaptability at different levels, thus providing a variety of mechanical properties and efficient distribution of loading. The network of interconnected fibers that they form also endows the ECM with a strong yet tunable mechanical resilience at very low solid volume fractions, thereby providing the necessary space for other ECM components and cells.^39^

Elastic fibers, composed of microfibrillar glycoproteins embedded in elastin, also exhibit a hierarchical structure.^40^ The formation of the elastic fibers starts with the synthesis of the microfibrillar glycoproteins, which act as a scaffold for the deposition of tropoelastin. Crosslinking of the elastin stabilizes the structure.^40^ In the media of arteries, elastic fibers form concentric layers of elastic lamellae, between which the vascular smooth muscle cells reside. The number of layers depends on the species, ranging from a few layers in mice to more than 50 layers in human arteries. Arteries are subjected to blood pressure, causing cyclic circumferential stretching, which induces a larger strain on the inner side of the arteries. The simultaneous extension and unfolding of the elastic lamellae compensate for this gradient of strain and maintain an even distribution of the circumferential stress throughout all the layers. Thus, the structural heterogeneity contributes to the maintenance of tissue homeostasis.^41^

The assembly and morphology of ECM fibrils strongly vary depending on the protein type as well as the tissues, organs, the mechanical functions they perform, and the species in which they are found.^42^ For example, collagen I fibrils are thicker (150–300 nm) than collagen III fibrils (25–100 nm). This difference corresponds to collagen type I predominating in organs bearing high tensile stress, such as bone and tendon, while collagen type III is a major structural component in internal hollow organs.^23^ Diameters of ECM fibrils in the dermis range from 60 to 120 nm and increase with depth, as mechanical stresses like stretch and compression are more prevalent deeper beneath the skin surface.^42,43^ The diameters of collagen fibers and elastin fibers in human pulmonary alveolar walls are both close to 1 μm and collagen fiber diameters slightly increase with age, as the alveolar wall size increases while its integrity has to be maintained against the mechanical stresses of respiration.^44^

The fibers of the ECM are not only important for tissue function (and mechanical properties, as will be discussed further below), but also define pores and spaces in the ECM through which cells can migrate and communicate. The pore size of ECM is typically in the micrometer range and varies across different tissues, organs, and species. Examples of the extremes include human corneal epithelial basement membrane and Descemet's membrane, with pore diameters of ∼92 nm and ∼38 nm,^45^ respectively, while human lateral meniscus has a pore diameter of 37–48 μm.^46^Table 2 summarizes the pore sizes of decellularized ECM in different species and tissues.

**Table 2:** Decellularized ECM: origin, pore diameter, fiber diameter, Young's modulus *E*, measurement method for the available data. Certain references originally measured pore dimension as area, and here such results are then converted to pore diameter with the assumption of perfect circle

Decellularized ECM originate from	Pore diameter	Fiber diameter	*E*		Measurement method for pore and fiber diameter	Measurement method for *E*	Ref.
			Macroscale	Nanoscale			
Mice lung	∼17.5 μm	∼1.13 μm	∼17 kPa	∼2.9 kPa	Scanning electron microscope (SEM)	Macroscale *E* is measured by macroindentation in a universal micromechanical system; nanoscale *E* is measured by AFM	47
Mice liver	∼13.0 μm	∼1.6 μm	∼20 kPa	∼5.6 kPa			
Mice kidney	∼10.7 μm	∼1.31 μm	∼22 kPa	∼6.1 kPa			
Mice spleen	∼10.0 μm	∼1.0 μm	∼40 kPa	∼3.6 kPa			
Mice ovary	∼1.4 μm	∼0.73 μm	∼19 kPa	∼6.5 kPa			
Porcine liver	∼10 μm	—	1.25–1.31 kPa		Silver staining of sections observed under inverted microscope	Compression test	48
Rat liver	—	0.58 ± 0.12 μm	97 ± 21.22 kPa		SEM	Tensile test	49
Mice salivary glands	10–30 μm	—	120 Pa		SEM	Micro-indentation tester	50

Another crucial structural characteristic of ECM fibers is their orientation, which is directly linked to the physiological function of the tissues. For example, blood vessels are composed of three layers (intima, media, and adventitia) with different fiber organizations in each layer, optimized to sustain shear stress induced by fluid flow as well as intramural pressure induced by the heartbeat.^51^ In articular cartilage, collagen fibers are oriented parallel to the surface in the superficial layer before transitioning to a perpendicular orientation in the deeper zone to resist wear and transmit the load.^52^ Tendon fibers are aligned parallel to the main axis to resist the tension.^53^

### 2.3. Mechanical properties

#### 2.3.1. Stiffness

The most frequently described mechanical parameter is stiffness.^54^ Stiffness is a measure of how much a material resists deformation and is mathematically defined as the ratio between the applied stress and the resulting strain. The stress and strain can be in different deformation modes (*e.g.*, tension, compression, shear, twist, bending), so while stiffness is often characterized by the elastic modulus in tension or compression (Young's modulus *E*), it can also refer to the bending or shear stiffness (shear modulus *G*).^55^ It should be mentioned that, in the biology literature, the term “stiffness” is sometimes used synonymously (but erroneously) or confused with other mechanical terms like “elasticity”, “compliance”, and “hardness”. These terms refer to distinct mechanical concepts, and readers are recommended to pay careful attention to how the quantity is defined and measured in the study.

The measured tissue stiffness depends on tissue hydration state, scale (from molecule to whole tissues), and test type (traction, bending, indentation compression, shear).^56^ Single collagen molecules and collagen fibrils (∅ 50–200 nm) have a Young's modulus of a few GPa.^57–59^ Computational studies estimated a decrease of Young's modulus from the single molecule level (∼6 GPa) to the fibril level (∼0.9 GPa), attributed to the staggered arrangement of collagen molecules in fibrils.^60^ At a larger scale, the measured elastic modulus of a single collagen fiber (∅ 325 ± 40 nm) was 100–360 MPa.^61^ Note that in some works describing the stiffness of ECM fibers such as collagen or fibrin, the terms “fibers” and “fibrils” are often used interchangeably to describe the fibril-bundle or fibril structures. Collagen fiber is not extensible, with a breaking strain of 10% for crosslinked fibril and Young's modulus in the range of 200–500 MPa. In contrast, a single fibrin fiber has an elastic modulus on the order of 1–10 MPa with a breaking strain of over 300%.^62,63^ For elastin, the elastic modulus is approximately 1 MPa with a breaking strain of 150%.^64^ The rather stiff and not extensible collagen fibers and soft and extensible fibrin and elastin correspond to their functions mentioned above: preventing excessive stretch and supporting elastic deformation, respectively.

At the tissue level, non-mineralized tissues exhibit a wide range of elastic moduli, from hundreds of Pa for the brain^65^ to several MPa for articular cartilage,^66^ while mineralized tissues such as bone and dentin can reach several GPa.^67,68^ The elastic and shear moduli of *ex vivo* tissues can be measured using mechanical testing machines through compression, tension, bending, or shear experiments. *In vivo* measurements of tissue require non-invasive methods: Boyer *et al*.^69^ measured Young's modulus of skin *in vivo* by their non-contact airflow system, while Pailler-Mattei *et al*.^70^ employed indentation experiments to exclude the elastic response of subcutaneous muscle, accurately estimating the skin's Young's modulus *in vivo*. Table 3 summarizes the influence of different measuring methods and different anatomical sites on the measured elastic modulus of bone (a hard, mineralized tissue) and skin (a soft, non-mineralized tissue), exemplifying the variations of measurement methods and the measured mechanical properties of human collagenous tissues.

**Table 3:** Examples of variations of elastic moduli reported for a hard, mineralized tissue (bone) and a soft, non-mineralized tissue (skin) depending on the type and the scale of the tests

Tissue	Elastic modulus	Method	Ref.
Bone cortical	17.4 GPa	Longitudinal compression	71
	9.6 GPa	Transversal compression	
	20 GPa	Nanoindentation	72
	8 GPa	AFM	73
Bone trabecular	18 MPa	Longitudinal compression	72
	6–10 MPa	Transversal compression	
Bone osteoid	27 kPa	AFM	74
Skin *in vivo*	20–1120 kPa	Torsion	75 and 76
	4–89 kPa	Indentation	70 and 77–79
	25–260 kPa	Suction	80,81
	0.3–20 MPa	Traction	82
Skin *ex vivo*	70–160 MPa	Traction	83 and 84
	0.1–322 kPa	AFM	85 and 86

#### 2.3.2. Viscoelasticity

Although stiffness is the most frequently reported and well-studied parameter, it is insufficient to capture the mechanical behavior of biological tissues. The biphasic structure (Fig. 1B) of the ECM contributes to the elastic and viscous nature of tissues (visco-elasticity).^87^ Viscoelasticity is a property related to the instantaneous elastic response to loading and energy storage and the time-dependent response to loading and energy dissipation during deformation. Viscoelastic properties of materials can be measured through creep tests (measurement of displacement under a constant load) and relaxation tests (measurement of stress under a constant displacement) (Fig. 1C – left). The relaxation time, which defines the characteristic time scale over which the resistance to deformation is relaxed, for native tissues is in the order of magnitude of tens to hundreds of seconds.^88^ Half-stress relaxation time (*τ*_1/2_) is defined as the time needed for relaxing half of the initial stress value, which can be used to analyze the stress relaxation response. Alternatively, viscoelasticity can also be characterized using dynamic mechanical analysis (DMA), where sinusoidal stress (or strain) is applied at a range of frequencies covering physiological and potentially pathophysiological loading conditions^89–92^ and the resulting strain (or stress) is measured.^93^ Storage modulus (*G*′) describes the in-phase stress–strain ratio, while loss modulus (*G*′′) describes the out-of-phase stress–strain ratio, indicating energy dissipation.

The viscoelastic response of ECM is critical in understanding ECM. For native soft biological tissues, the ratio of loss modulus to storage modulus (tan *δ*) ranges from 0.1 to 0.2 when measured at 1 Hz.^88^ At higher shear frequencies (50–500 Hz), soft tissues such as the brain, liver, and muscles behave more like liquids, with tan *δ* values ranging from 0.3 to 0.7^21^ (Fig. 1C). The energy dissipation during loading occurs through three main mechanisms:^88^ the unbinding or breaking of non-covalent weak bonds among fibers such as collagen,^94^ the release of entanglements among polymer fibers, and the unfolding of protein fibers such as fibrin.^95^

#### 2.3.3. Linearity and nonlinearity

Stiffness and viscoelastic properties of biological tissues are usually reported as single values obtained at relatively low stresses or strains, *i.e.*, in the “linear regime”. In the context of a stress–strain plot, linearity is depicted by a straight line through the origin, representing a direct proportionality between stress *σ* and strain *γ*. Conversely, deviations from this straight line in the stress–strain plot denote nonlinearity, and in fact, many tissues and biological samples exhibit nonlinear behaviors.^96^

Strain-stiffening refers to a mechanical property characterized by a material becoming stiffer or exhibiting increased resistance to deformation as it is increasingly strained. Besides the stress–strain plot, the differential modulus *K*′ as a function of applied stress *σ* is also used to describe strain-stiffening (Fig. 1C – right). *K*′ can be calculated by δ*σ*/δ*γ*. The material exhibits a linear response at low stress levels, where *K*′ is almost constant and equals the plateau modulus *G*_0_. As the stress *σ* increases beyond a critical threshold *σ*_c_, the material enters a non-linear regime where *K*′ can be described as *K*′ = *σ*^*m*^, with *m* being the stiffening index. This critical stress *σ*_c_ and the corresponding critical strain *γ*_c_ mark the end of the linear regime and the onset of strain-stiffening. Collagen-based tissues are representative examples of strain-stiffening, such as vessels, tendons, and ligaments, which are primarily composed of collagen fibers^51,53^ (Fig. 1C). On the contrary, strain softening describes a phenomenon wherein a material exhibits decreased resistance to deformation with increasing strain. For instance, during shear loading, tissues such as the brain^97^ and liver^98^ exhibit shear-softening.

#### 2.3.4. Poroelasticity

Another mechanical property related to both nonlinearity and viscoelasticity, but distinct from viscoelasticity, is poroelasticity, which describes the change in volume under load due to fluid flow through the fibers and out of the network,^99^ similarly to a sponge losing its water under compression. Poroelasticity leads to greater energy dissipation compared to the often-assumed constant-volume condition.^88,100^ Under shear loading, non-poroelastic materials like rubber and polyacrylamide exhibit positive normal stress, while poroelastic fibrous biopolymers display negative normal stress due to fluid efflux, particularly suppressed at higher frequencies.^101^ Poroelasticity introduces nonlinearity in normal stress, which is also influenced by the shear modulus (*G*). However, the direct relationship between poroelasticity and nonlinear *G* has yet to be extensively explored, indicating a need for more detailed research in this area.

### 2.4. ECM as a dynamic environment

The term “dynamic” can assume different meanings depending on the subject matter. Materials are often characterized as “dynamic” when they exhibit time-scale-dependent viscoelastic behavior/property under mechanical loading, in which case “dynamic” refers to the time-dependent application of stress or strain and the response of the material. In a physiological context, “dynamic” refers to the inherently continuous remodeling of the ECM, which constantly undergoes remodeling to maintain mechanical homeostasis. Cells degrade old ECM components and secrete new ones, and ECM fibers undergo reorganization and crosslinking.^102^ This dynamic remodeling enables the ECM to adapt in response to mechanical or biochemical cues. For example, in the ECM of bone,^103^ tendon, and skeletal muscle,^104^ matrix deposition and degradation depend on mechanical loading. Additionally, certain mechanisms, such as osteocyte differentiation^105^ and capillary formation,^106^ depend on the matrix metalloproteinase (MMP)-mediated degradation of the existing matrix. This balance can be altered during aging or disease. The ECM dynamism *in vivo* is influenced by the stabilization of collagen and elastin through covalent bonds formed by lysyl oxidase (LOX) and lysyl oxidase-like (LOXL), as well as by advanced glycation end products, which contribute to increased matrix stiffness.^26^ Notably, LOX is crucial for the regeneration of ECM mechanical strength after injury.^107^ Advanced glycation end products accumulate through aging, resulting in increased stiffness, and reduced viscoelasticity.^108^

Some pathologies can alter ECM composition, structure, and mechanical properties, which are inextricably linked.^87^ Fibrosis, a disease accounting for 45% of deaths in industrialized nations,^109^ is marked by excessive ECM deposition, which in turn stimulates fibroblasts to increase ECM production, thus activating a positive feedback loop.^110^ Fibrosis is associated with an overexpression of transglutaminases and LOXs, as well as advanced glycation end products, which increase the extent of collagen and elastin fiber crosslinking in the tissue, thereby increasing the stiffness and reducing the viscosity of the ECM.^35^ Additionally, fibrosis is also associated with an increased HA content,^111^ which enhances the swelling pressure, induces isotropic stretching of the collagen fibers, and diminishes anisotropic alignment under tensile stresses, further leading to enhanced isotropic stiffening and reduced anisotropic strain-stiffening of collagen fibers. This leads to the tissue generally becoming more mechanically linear^36^ and diminishes the effectiveness of long-distance cell force transmission.^112^ The ECM structure of the fibrotic tissue also changes: in conditions such as idiopathic pulmonary fibrosis, the collagen in ECM is characterized as more highly anisotropic compared to nonfibrotic lung.^113^ Cancer impacts the ECM structural properties and mechanical properties such as stiffness and viscoelasticity.^114^ While some cancers such as breast scirrhous carcinoma, prostate cancer, and thyroid cancer increase ECM stiffness, others like intraductal and papillary carcinoma soften it.^42^ ECM stiffness affects cancer cell morphology, proliferation, invasion, and therapeutic efficacy. However, these effects depend on the cancer type and are not universally applicable, indicating that while ECM stiffness is a promising target for cancer treatment, it necessitates further investigation.^115^ The alignment of ECM fibers perpendicular to the tumor boundary not only increases tissue stiffness but also forms structures that act as routes that facilitate cancer cell migration.^116,117^ Cancer-associated fibroblasts are one of the sources that produce and direct the assembly of an anisotropic network of collagen I, which promotes tumor cell spread from the primary tumor site.^118^ Keloid fibroblasts also induce ECM anisotropy.^119^

## 3. Engineering hydrogels

As we have shown in the previous section, native ECM offers a complex mechanical and biochemical environment for cells. To better understand the role of each cue on cell behavior, it is useful to isolate each cue separately using *in vitro* models. Given the three-dimensional, soft, viscoelastic, and biphasic nature of the ECM, hydrogels have been particularly useful in recapitulating cellular environments in a laboratory setting and in studying the cell–ECM interface. Hydrogels are defined as polymer chain networks that retain over 90% of volume as water within the interstitial spaces between polymer chains.^120^ Their fabrication relies on the transition of a liquid precursor solution into a gel, during which the hydrogel components are assembled by physical crosslinking or chemical crosslinking.^121^ Most peptide- or protein-based hydrogel networks are created through self-assembly *via* physical crosslinking methods. For more details about synthesizing hydrogels by physical or chemical crosslinking, readers are referred to the review by Hoffman.^122^ A key advantage of hydrogels is their tunability, allowing various structural and compositional properties to be adjusted to modify their mechanical and structural characteristics. As we will discuss in the upcoming sections, however, decoupling these parameters is complicated as structure and mechanics influence each other.

### 3.1. Polymer types and ligands

The biochemical signatures of the native ECM can be replicated by selecting appropriate polymers and ligands in hydrogels. These polymer networks can originate from either natural or synthetic sources. Some natural polymers are derived from the ECM and inherently contain binding peptides, while others require functionalization to facilitate cell adhesion. Common biological polymers used as hydrogels for cell culture include collagen, gelatin (the denatured form of collagen), and fibrin. Another widely used natural polymer is Matrigel, which is derived from mouse sarcoma and primarily composed of laminin, collagen IV, and entactin, along with smaller amounts of structural proteins and various growth factors. It should be noted that, due to the presence of multiple proteins and growth factors, Matrigel creates a poorly defined chemical environment, which can affect the reproducibility of studies.^54,123^

Polymers not derived from natural ECM, such as non-mammalian polysaccharides^124^ (*e.g.*, alginate, dextran) or synthetic polymers^125^ (*e.g.*, polyethylene glycol (PEG),^126^ polyvinyl alcohol (PVA),^127^ ureido-pyrimidinone (UPy)^128^), require functionalization to enable cell binding. While various peptides can be used for functionalization, RGD is the most common. Additionally, polymers not derived from the ECM cannot be naturally degraded by cells. This limitation can be overcome by incorporating MMP-degradable crosslinkers. For example, PEG gels can be crosslinked with MMP-sensitive bonds to facilitate cell-mediated degradation.^129–132^

The use of synthetic polymers has improved our knowledge of the effect of the biochemical environment on cell behavior. Notably, the functionalization of polymers enables researchers to vary ligand density independently of polymer concentrations^133,134^ and to vary ligand type independently of mechanical properties.^135^ This revealed that both ligand type^135^ and ligand density^134^ influence cell phenotype and traction forces. For example, valvular interstitial cells embedded in PEG remained round in the presence of IKVAV peptides but were able to spread in the presence of RGD.^135^ Increasing the concentration of RGD peptide in PEG hydrogels from 0.5 mM to 2 mM enabled the formation of vinculin complexes at the periphery of mouse embryonic fibroblasts, which resulted in larger strains and stresses on the surrounding matrix.^134^

Synthetic hydrogels also enable studies on the importance of matrix degradation for cell spreading and differentiation. For example, the differentiation of osteoblasts to mature osteocytes requires dendrite extension, which is enhanced in PEG gel containing MMP-sensitive crosslinkers.^130^ Furthermore, degradability is of major importance for regenerative medicine as it enhances cellular invasion from neighboring tissues^136^ and neo-tissue formation.^131,137^

### 3.2. Structure

#### 3.2.1. Porosity

Similar to the ECM, hydrogels are porous and biphasic. The solid polymer phase, or solid fibers, functions as a framework and provides physical boundary conditions for cells, while the liquid phase allows nutrient transport and diffusion. Porous materials are described in terms of three parameters: permeability, porosity, and pore size. Permeability describes how easy it is for molecules to diffuse through and for fluid to pass through the pores of the hydrogel. It is quantified by the fluid flow velocity under a controlled pressure difference, typically exerted by the weight of the fluid or an external pump, and is expressed as the flow rate per unit area per unit pressure drop.^138–140^ Porosity is defined as the ratio of the volume of pores to the total volume of the gel.^141^ Pore size refers to the characteristic length scale of the pores. In this review, two distinct definitions of pore size are described: (1) intrinsic pore size (or mesh size), referring to the microscopic pores formed by the polymer network, defined as the distance between crosslink points or solid fibers;^54^ and (2) macropore size, referring to voids introduced to create macroporous structures.^142,143^ Intrinsic pore size is discussed in this section, while macropore size is covered in section 3.6.

Pore size can be imaged using scanning electron microscopy (SEM), though this technique introduces a bias as it requires drying the hydrogels, which can distort the microstructure.^121^ Other imaging techniques such as confocal microscopy associated with image post-processing^144^ or fluorescence recovery after photobleaching (FRAP) can be used to estimate pore size in hydrated states.^121^ Mean pore size or mean pore diameter can be represented by permeability and can be determined by diffusion-driven transport of FITC-labeled dextran (Table 4). Maximum pore size can be estimated by DNA electrophoresis.^145^ The polymer network of hydrogels creates molecular-size openings in the nanometer scale for most non-fibrous synthetic hydrogels, and micrometer scale in fibrous hydrogels such as collagen and fibrin.^54,146^ The range of pore sizes depends on the experimental conditions, for example, concentrations of polymers and crosslinkers, and temperature.

**Table 4:** Hydrogel: polymer component, pore diameter, fibril diameter, Young's modulus *E*, storage modulus *G*′, loss modulus *G*′′, measurement method for pore and fibril diameter, measurement method for mechanical properties, reference. Certain references originally measured pore dimension as area, and here such results are then converted to pore diameter with the assumption of perfect circle. The data are mean value unless otherwise specified. Data of which the exact values are not specifically listed in the text of references were extracted from the figures in the references using an online tool^147^

Polymer	Pore diameter	Fibril diameter	*E*	*G*′	*G*”	Measurement method for pore and fibril diameter	Measurement method for mechanical properties (*E*, *G*′, *G*′′)	Ref.
Collagen-I 1 mg mL^−1^, gelation temperature 22 °C	11 μm	—	—	∼0.7 Pa	—	Confocal reflectance microscopy images	Rheology	148
Collagen-I 1 mg mL^−1^, gelation temperaturen 37 °C	∼5 μm			∼2 Pa				
Collagen 1 mg mL^−1^	5.27 μm	126.83 nm	1.40 kPa	—	—	Pore size: confocal laser scanning microscope of fluorescence image of collagen matrix; collagen fibrils: scanning electron microscopy	Effective elastic modulus was measured by a custom-made indentation apparatus	149
Collagen 1 mg mL^−1^ + Na_2_SO_4_ 0.025 mol L^−1^	4.73 μm	142.00 nm	1.65 kPa					
Collagen 1 mg mL^−1^ + Na_2_SO_4_ 0.10 mol L^−1^	6.57 μm	161.66 nm	1.73 kPa					
Collagen 1 mg mL^−1^ + Na_2_SO_4_ 0.25 mol L^−1^	6.84 μm	201.85 nm	2.01 kPa					
Collagen-I 1 mg mL^−1^	—	40 ± 3.1 nm	—	2.63 ± 0.86 kPa	—	Aomic force microscope (AFM) image	Rheology	150
Collagen-I 1.2 mg mL^−1^ (from rat tail)	—	—	∼0.36 kPa	119 ± 63 Pa	13 ± 6 Pa	—	Macroindentation by universal micromechanical system; rheology	47
Collagen 1.5 mg mL^−1^, pH = 7	4.5 μm	—	—	6.1 Pa	—	Bubble analysis from confocal reflectance images	Oscillatory shear rheology	151
Collagen-I 1.5 mg mL^−1^; (from pure rat tail monomers)	7.7 ± 1.4 μm	—	63.0 ± 48.5 Pa	—	—	Confocal laser scanning microscopy	AFM	152
Collagen-I 1.5 mg mL^−1^; (rat tail monomers :bovine skin monomers = 1 : 2)	7.3 ± 0.7 μm		101.2 ± 68.5 Pa					
Collagen-I 1.5 mg mL^−1^; (from pure bovine skin monomers)	6.9 ± 1.5 μm		76.1 Pa					
Collagen-I 2 mg mL^−1^	1.051 μm	1.004 μm	0.208 kPa	—	—	Confocal reflectance microscopy & ImageJ	Indenter (pre-loaded compression followed by retraction until separation, result calculated from slope of experimentally measured force-indentation adjusted area (as AFM))	153
Collagen 2 mg mL^−1^	17.48 μm	∼850 nm	49.59 Pa	—	—	Confocal microscope	AFM	154
Collagen 2.5 mg mL^−1^ pH = 9	10.9 μm	—	—	61.0 Pa	—	Bubble analysis from confocal reflectance images	Oscillatory shear rheology	151
Collagen 2.5 mg mL^−1^ pH = 7	11.4 μm			20.1 Pa				
Collagen 2.5 mg mL^−1^ pH = 6	13.1 μm			5.7 Pa				
Collagen 2.5 mg mL ^−1^	3.06 μm	0.73 μm	90.1 ± 3.0 Pa	—	—	Confocal laser scanning microscope	Colloidal probe force spectroscopy	155
Collagen 2.5 mg mL^−1^, with covalently intramolecular crosslinking of fibrils by 20 mM EDC (1-ethyl-3-(3-dimethyl-aminopropyl)-carbodiimide)	3.75 μm	0.72 μm	163.6 ± 6.6 Pa					
Collagen-I 3 mg mL^−1^ (from rat tail)	1.13 μm	0.184 μm	2.16 kPa	—	—	Confocal reflectance microscopy	Indentation	156
Collagen-I 3 mg mL^−1^	1.70 μm	—	3.15 kPa	—	—	Confocal reflectance microscopy	Indentation	157
Collagen 3 mg mL^−1^	13.0 ± 13.8 μm	—	—	0.05 kPa	—	Scanning electron microscope (SEM)	Rheology	129
Collagen 3 mg mL^−1^, in buffer solution of pH 6.5	—	86.04 nm	—	18.94 Pa	3.84 Pa	Cryo-SEM imaging	Rheology	158
Collagen 3 mg mL^−1^, in buffer solution of pH 7		138.21 nm		31.82 Pa	6.22 Pa			
Collagen 3 mg mL^−1^, in buffer solution of pH 7.4		71.67 nm		23.56 Pa	4.35 Pa			
Collagen 3 mg mL^−1^, in buffer solution of pH 7.8		71.62 nm		28.32 Pa	5.25 Pa			
Collagen-I 3 mg mL^−1^ (from pure rat tail monomers)	5.8 ± 0.4 μm	—	292.9 ± 321.9 Pa	—	—	Confocal laser scanning microscopy	AFM	152
Collagen-I 3 mg mL^−1^ (rat tail monomers : bovine skin monomers = 1 : 2)	5.9 ± 1.3 μm		326.2 ± 260.1 Pa					
Collagen-I 3 mg mL^−1^ (from pure bovine skin monomers)	5.2 ± 1.6 μm		141.5 Pa					
Collagen-I 4 mg mL^−1^, gelation temperature 22 °C	∼7 μm	—	—	∼70 Pa	—	Confocal reflectance microscopy	Rheology	148
Collagen-I 4 mg mL^−1^, gelation temperature 37 °C	∼2.5 μm			∼110 Pa				
Collagen 4 mg mL^−1^ (bovine collagen-I (97%) and collagen-III (3%)), pH = 7	1.3 μm	—	—	74.4 Pa	—	Bubble analysis from confocal reflectance images	Oscillatory shear rheology	151
Collagen 4 mg mL^−1^	∼1.4 μm	∼0.9 μm	—	101 Pa	—	FEI NOVA nanoscanning electron microscopy	Rheology	159
Collagen 4 mg mL^−1^	10.82 μm	∼850 nm	64.39Pa	—	—	Confocal microscope	Elastic modulus by AFM	154
Collagen 4 mg mL^−1^, gelation temperature 37 °C	1.17 μm	—	—	240.26 Pa	—	Confocal laser scanning microscope	Rheology	160
Collagen 4 mg mL^−1^, gelation temperature 15 °C	4.37 μm			332.56 Pa				
Collagen 6 mg mL^−1^, gelation temperature 37 °C	148 nm	124 nm	0.783 kPa (DMTA), 0.458 kPa (AFM)	252 Pa (shear moduli *G*)		SEM	Dynamic mechanical thermal analysis (DMTA) & AFM for static compression (omit poroelastic effect); rheology for shear moduli	161
Collagen 6 mg mL^−1^, gelation temperature starts from 4 °C and heat to 37 °C	182 nm	163 nm	0.718 kPa (DMTA), 0.464 kPa (AFM)	2064 Pa (shear moduli *G*)				
Collagen 6 mg mL^−1^	7.22 μm	∼850 nm	100.15 Pa	—	—	Confocal microscope	Elastic modulus by AFM	154
Collagen 6 mg mL^−1^, gelation temperature 37 °C	1.24 μm	—	—	397.33 Pa	—	Confocal laser scanning microscope	Rheology	160
Collagen 6 mg mL^−1^, gelation temperature 15 °C	4.12 μm			694.47 Pa				
Gelatin 6.0 wt%, altering intrafibrillar crosslinking by various methacryloyl groups and/or photoirradiation time	93.7 nm	28.0–30.2 nm	0.5–11.2 kPa	1.0 kPa–2.1 kPa	0.006–0.02 kPa	Diffusion-driven transport of 70 kDa FITC-dextran molecules	Cyclic compression experiments using a Mach-1 Mechanical tester; rheology	162
HA 30 mg mL^−1^, crosslinked	∼ 6 μm	—	—	537 Pa	—	FEI NOVA nanoscanning electron microscopy	Rheology	159
Collagen-I 2 mg mL^−1^ + HA 0.2 mg mL^−1^, with molecular weight 100–150 kDa (LMW)	1.134 μm	1.156 μm	0.187 kPa	—	—	Confocal reflectance microscopy & ImageJ	Indenter (pre-loaded compression followed by retraction until separation, result calculated from slope of experimentally measured force-indentation adjusted area (as AFM))	153
Collagen-I 2 mg mL^−1^ + HA 0.2 mg mL^−1^, with molecular weight 1.01–1.8 MDa (HMW)	1.159 μm	1.187 μm	0.215 kPa					
Collagen-I 2 mg mL^−1^ + HA 2 mg mL^−1^ LMV	1.158 μm	1.419 μm	0.404 kPa					
Collagen-I 2 mg mL^−1^ + HA 2 mg mL^−1^ HMV	1.207 μm	1.631 μm	0.628 kPa					
Collagen-I 4 mg mL^−1^ + HA 4 mg mL^−1^ LMV	1.195 μm	1.685 μm	0.627 kPa					
Collagen-I 4 mg mL^−1^ + HA 4 mg mL^−1^ HMV	1.165 μm	2.049 μm	0.727 kPa					
Collagen 3 mg mL^−1^ + HA 1 mg mL^−1^	1.92 μm	—	6.44 kPa	—	—	Confocal reflectance microscopy	Indentation	157
Collagen 4 mg mL^−1^ + HA 30 mg mL^−1^	∼1.7 μm	∼0.9 μm	—	1676 Pa	—	FEI NOVA nanoscanning electron microscopy	Rheology	159
Methacrylated hyaluronic acid (HA-MA) 1 wt% + metacrylate gelatin (GelMA) 0.5% (w/w); with high degree of methacrylation of HA (specific ratios not stated)	12.90 μm	—	18.54 kPa	1187 Pa	246.64 Pa	Field-emission SEM	Universal testing machine for compressive moduli; rheology for *G*′ and *G*′′	163
Methacrylated hyaluronic acid (HA-MA) 1 wt% + metacrylate gelatin (GelMA) 0.5% (w/w); with middle degree of methacrylation of HA	14.43 μm		13.43 kPa	308 Pa	100.17 Pa			
Methacrylated hyaluronic acid (HA-MA) 1 wt% + metacrylate gelatin (GelMA) 0.5% (w/w); with low degree of methacrylation of HA	23.17 μm		6.05 kPa	118 Pa	47.37 Pa			
GelMA 5% (w/v)	Measured after swollen of 1/7/14 days: 310/265/301 μm	—	5.2 kPa	—	Stress relaxation percentage 8.2%	SEM	Uniaxial compression for elastic modulus and stress relaxation for *G*′′	164
GelMA 10% (w/v)	192/124/199 μm		55.6 kPa		5.5%			
GelMA 15% (w/v)	163/182/229 μm		161.1 kPa		6.3%			
GelMA 5 wt%	7.4 μm	—	0.034 kPa	—	—	SEM	Rheology for measurement of *G*′ and *G*′′ (data not displayed for both) and calculation of *E* 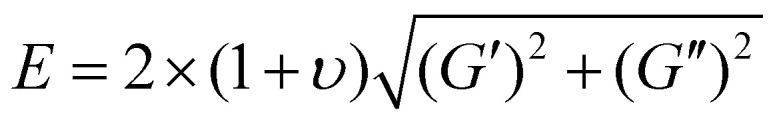 *υ* = 0.5	165
GelMA 10 wt%	7.3 μm		1.82 kPa					
GelMA 15 wt%	2.6 μm		2.68 kPa					
Poly (ethylene glycol) diacrylate (PEGDA)-GelMA, 7.5 : 1 wt%	9.1 μm		6.18 kPa					
Poly (ethylene glycol) diacrylate (PEGDA)-GelMA, 1 : 10 wt%	3.6 μm		2.01 kPa					
Imine-cross-linked cellulose nanocrystals (CNCs) and gelatin, *C*_CNC_-to-*C*_gel_ = 0.5, *C*_total_ = 1.0 wt%	1.2 μm	107 ± 35 nm	—	4 Pa	0.4 Pa	Pore size by Darcy permeability of hydrogels; diameter of CNC by transmission electron microscopy (TEM)	Rheology	166
Imine-cross-linked cellulose nanocrystals (CNCs) and gelatin, *C*_CNC_-to-*C*_gel_ = 0.5, *C*_total_ = 6.0 wt%	57 nm	31 ± 14 nm		254 Pa	10.5 Pa			
Gelatin 1 wt% + alginate 3 wt%	0.69 μm	—	83.16 kPa	—	—	SEM	Compression by electronic universal material testing machine	167
Gelatin 1 wt% + alginate 3 wt% + 2 wt% bioactive nanoparticles (BNPs)	0.60 μm		67.89 kPa					
Gelatin 1 wt% + alginate 3 wt% + 5 wt% BNPs	0.57 μm		63.99 kPa					
Gelatin 2 wt% + alginate 5 wt%	0.57 μm		136.59 kPa					
Gelatin 2 wt% + alginate 5 wt% + 2 wt% BNPs	0.55 μm		146.767 kPa					
Gelatin 2 wt% + alginate 5 wt% + 5 wt% BNPs	043 μm		152.906 kPa					
Alginate 1% w/v + 100 mM Ca^2+^; sank into 0.3% chitosan solution after gelation	101.4 ± 48.1 μm	—	42. 8 ± 3.1 kPa	—	—	SEM	Compression tests	168
Alginate 3% w/v + 10 mM CaCl_2_	23.3 ± 1.2 nm	—	—	628 ± 101 Pa	—	Mesh size was calculated based on rheological measurement: 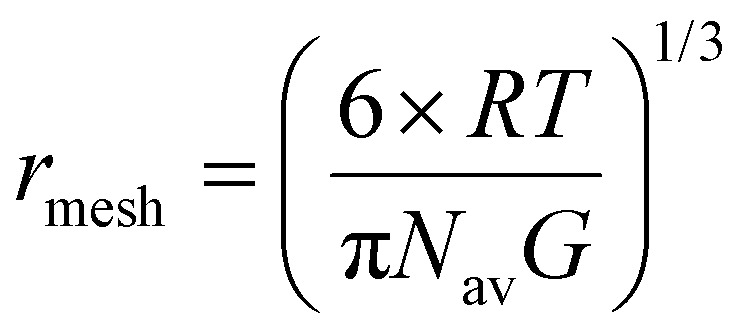 , where *R* is the gas constant, *T* is the absolute temperature, *N*_av_ is Avogadro's number, and *G* is the storage modulus	Rheology	169
Alginate 3% w/v + 40 mM CaCl_2_	13.7 ± 1.0 nm			3144 ± 686 Pa				
Alginate 1% w/v + collagen 0.125% w/v + 100 mM Ca^2+^; sank into 0.3% chitosan solution after gelation	93.7 ± 46.7 μm	—	55.3 ± 6.6 kPa	—	—	SEM	Compression tests	168
Phenolated alginate 1.2% w/v + nano-hydroxyapatite 1% w/w	486 ± 16.72 μm	—	12.8 kPa	—	—	SEM	Tensile machine	170
Phenolated alginate 1.2% w/v + collagen-I 0.5% w/v + nano-hydroxyapatite 1% w/w	470.5 ± 17.84 μm		15.6 kPa					
Alginate 5 mg mL^−1^ + collagen 1.33 mg mL^−1^ + CaCl_2_ 5.0 mM	45.9 nm	54.9 ± 10.8 nm—	0.14 ± 0.01 kPa—	46.9 Pa	—	Pore diameter is calculated by diffusive transport of FITC labeled dextran; SEM for fiber diameter	Rheology for measurement of *G*′ and *G*′′ (data not displayed for *G*′′) and calculation of *E* 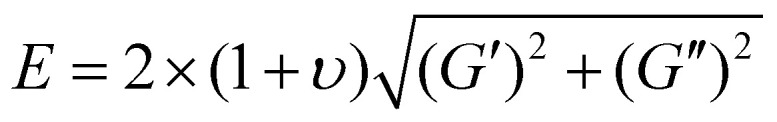 *υ* = 0.5	171
Alginate 5 mg mL^−1^ + collagen 1.33 mg mL^−1^ + CaCl_2_ 7.5 mM	55.1 nm			108 Pa				
Alginate 5 mg mL^−1^ + collagen 1.33 mg mL^−1^ + CaCl_2_ 10 mM	40.4 nm			374 Pa				
Alginate 5 mg mL^−1^ + collagen 1.33 mg mL^−1^ + CaCl_2_ 12.5 mM	42.5 nm			665 Pa				
Alginate 5 mg mL^−1^ + collagen 1.33 mg mL^−1^ + CaCl_2_ 15 mM	50.1 nm	59.7 ± 12.3 nm	2.7 ± 0.39 kPa	902 Pa				
Fibrin, 30 mg mL^−1^	32.2 ± 3.8 nm	—	—	250 ± 74Pa	—	Mesh size was calculated based on rheological measurement: 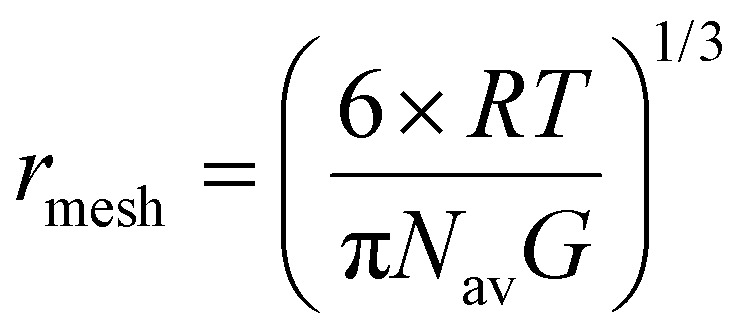 , where *R* is the gas constant, *T* is the absolute temperature, *N*_av_ is Avogadro's number, and *G* is the storage modulus	Rheology	169
IPN (fibrin 30 mg mL^−1^ + alginate 3% w/v) + 10 mM CaCl_2_	21.3 ± 1.7 nm			831 ± 205 Pa				
IPN (fibrin 30 mg mL^−1^ + alginate 3% w/v) + 40 mM CaCl_2_	15.1 ± 2.3 nm			2588 ± 1266 Pa				
Fibrin 10 mg mL^−1^ + collagen 2.5 mg mL^−1^ + thrombin 2.5 U ml^−1^	0.4085 μm (median)	—	2886 Pa	—	—	SEM	AFM	172
Matrigel 50% v/v in DMEM	3.234 μm	—	—	8.92 Pa	—	Quick-freeze, deep-Etch electron microscopy	Rheology	173
Matrigel 67% v/v in DMEM	3.115 μm			17.76 Pa				
Matrigel 75% v/v in DMEM	2.986 μm			32.33 Pa				
Matrigel 25% v/v + alginate 16 mg mL^−1^ + CaCO_3_ 174 mM	51.2 ± 2.8 μm	—	2.66 ± 0.84 kPa	0.85 kPa	0.18 kPa	SEM	Rheology for measurement of *G*′ and *G*′′ and calculation of *E* 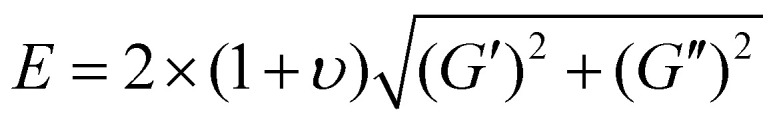 *υ* = 0.5	174
Matrigel 25% v/v + alginate 28 mg mL^−1^ + CaCO_3_ 453 mM	38.4 ± 15.9 μm		8.98 ± 1.29 kPa	2.90 kPa	0.58 kPa			
Matrigel 25% v/v + alginate 40 mg mL^−1^ + CaCO_3_ 500 mM	32.3 ± 9.1 μm		18.27 ± 3.17 kPa	6.02 kPa	0.67 kPa			
Decellularized porcine arterial ECM-derived hydrogel 30 mg mL^−1^ (after lyophilized, liquefied, and enzymatic digestion)	Distribution: <5 μm (∼50%); 5–50 μm (∼50%)	Distribution: 1–2.5 μm (∼67%); 2.5–5 μm (∼30%); <1 μm (∼1.5%); 5–10 μm (∼1.5%)	3.47 ± 1.3 kPa (spatial elastic modulus)	∼8 kPa	1.4–1.8 kPa	SEM	Spatial elastic modulus by AFM; rheology	175
Chitosan 4% w/w	4.96 nm	—	110.51 ± 19.33 kPa	—	—	SEM	Tensile stress–strain tests	176
The tetramer peptide IIZK (Ac-Ile-Ile-Cha-Lys-NH2) 1 mg mL^−1^ in 1× PBS	—	9 nm–16 nm (no difference for the two concentrations)	—	7.3 kPa	1.1 kPa	SEM and Cryo-TEM	Rheology	177
The tetramer peptide IIZK 10 mg mL^−1^ in 1× PBS				139.7 kPa	12.2 kPa			
IKVAV appended with 9-Fluorenylmethoxycarbonyl modification (Fmoc IKVAV) 20 mM; (YIGSR and IKVAV are laminin mimetic pentapeptides)	—	30 ± 5 nm (AFM); 15 ± 2.2 nm (TEM)	—	∼267 ± 47 Pa	—	Fiber diameter by AFM or TEM	Rheology	178
Fmoc YIGSR 20 mM		20 ± 6 nm (AFM); 11.5 ± 1.2 nm (TEM)		∼674 ± 100 Pa				
Fmoc IKVAV 10 mM + Fmoc YIGSR 10 mM		85 ± 14 nm mix with 32 ± 6.5 nm (AFM); 28.3 ± 2.5 nm mix with 9.5 ± 1.9 nm (TEM)		∼937 ± 124 Pa				
4-arm PEG maleimide 5 mM (10 kDA) + MMP-sensitive crosslinker 10 mM	Median pore diameter 13.3 ± 14.1 μm	—	—	1.4 ± 0.3 kPa	—	SEM	Rheology	129
4-arm PEG maleimide 5 mM (10 kDA) + 4-arm PEG thiol 3.33 mM (10 kDA) + MMP-sensitive crosslinker 3.33 mM	10.7 ± 11.0 μm			7.9 ± 0.7 kPa				

Collagen mesh size can be predicted by the equation from Jansen *et al*.:^179^*ξ* = (1/*ρ*_l_)^0.5^, with *ξ* the average mesh size, *ρ*_l_ the length of collagen fibers per unit volume defined as *ρ*_l_ = *c*_p_/*μ*, with *c*_p_ the concentration of collagen, and *μ* the average mass–length ratio of the fibers. Fiber diameter *d* and *μ* both decrease with increasing *c*_p_ or increasing temperature. These parameters can be calculated using the turbidity–wavelength relationship of collagen fibrous gels, as measured by light scattering. However, it is important to note that collagen sourced from different species and different extraction and purification processes have different polymerization times, mechanical properties, and structures, even under the same polymerization conditions. The most prevalent collagen I sources are bovine dermis,^180^ murine tendon,^180^ and porcine skin.^181^ Collagen can be extracted through acid or enzymatic (pepsin) treatment. Pepsin-treated collagen self-assembles into multimeric fibrils more slowly, resulting in a larger mesh size (3–5 μm) and longer fibrils. In contrast, acid-extracted collagen, even with the same protein content, forms smaller pores (1–2 μm) and shorter fibrils.^182^

Similarly to collagen, fibrin pore size depends on fibrinogen and thrombin concentrations. Wufsus *et al*.^183^ estimated the fibrin network mesh size *ξ* from the square root of hydraulic permeability *K*_s_ (*ξ* = *K*_s_^1/2^) or from measurements of shear modulus 
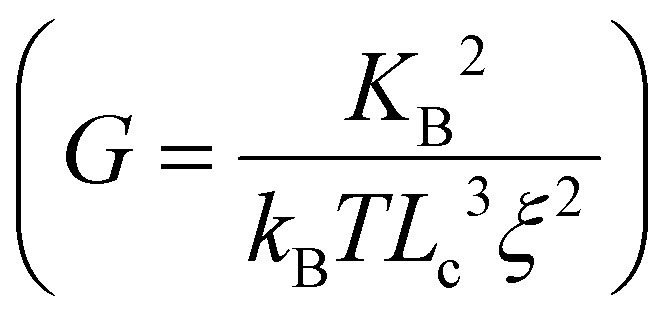
, and the results of the two methods had good agreement. The mesh size decreased from 300–400 nm to 20–30 nm when the fibrinogen concentration increased from 3 mg mL^−1^ to 100 mg mL^−1^, and the most obvious change occurred over the range of 3–30 mg mL^−1^. These findings were confirmed by other studies.^184–186^

**Fig. 2:**
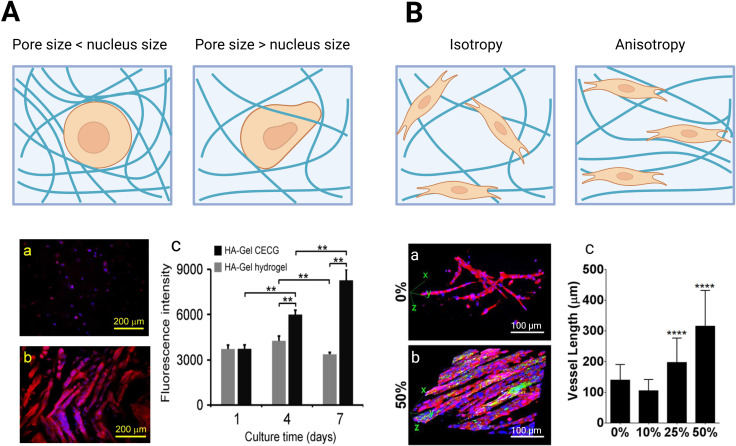
Relation between hydrogel structure and cell behavior. (A) When the pore size is smaller than the nucleus size, cells are confined within the pore and can't migrate or spread. When the pore size is larger than the nucleus, cells can squeeze in and elongate. Human mesenchymal stromal cells were encapsulated in either a hyaluronic gel (HA-gel Hydrogel) with a nanopore size (a) or a cell encapsulatable cryogel (HA-gel CECG) with macropores (b). Cells in the HA-gel expressed a low level of actin, while cells in the HA-gel CECG showed a high level of actin and were able to spread. The increased pore size also enabled cell growth, as indicated by the presto blue assay (c) (reproduced from ref. 189 with permission from IOP Publishing, copyright 2019). (B) Fiber orientation can guide cell orientation. Endothelial cells were embedded in collagen gel with preferential or aligned collagen fibers. When fibers were randomly oriented, cells were also randomly oriented (a). When fibers were aligned in a preferential direction (anisotropy), cells aligned in that direction (b) and formed longer vessels (c) (reproduced from ref. 190 with permission from American Chemical Society (ACS), copyright 2018).

Pore size has a direct effect on cells by inducing mechanical confinement, but also an indirect effect by influencing nutrient diffusion (Fig. 2A).^121^ The intrinsic pore size of hydrogels has been shown to significantly influence the migratory behavior of cells and alter cellular fate decisions. The nanometer scale meshes of synthetic polymer networks confine cells, making cell migration or morphological change nearly impossible without deforming or degrading the hydrogel.^54^ Rigid matrices with mesh sizes less than 10% of nuclear cross section (≤7 μm^2^) were reported to be able to block the squeezing through of cell nuclei.^187^ Frequent squeezing through small intrinsic pores comparable in size to a cell or its nucleus and characterized by high stiffness may result in DNA damage.^188^

#### 3.2.2. Fiber orientation

Another important hydrogel structural property is fiber orientation. Methods to achieve controlled fiber orientation have focused mainly on collagen I hydrogels. Though several methods exist to align collagen, we only included those compatible with cell embedding. Applying strains during or after collagen crosslinking is common. Pre-alignment of collagen fibers can be achieved by casting and crosslinking collagen on pre-stretched PDMS molds that are then released,^190^ by magnetic field-induced flow of magnetic beads,^191,192^ or by applying shear stress with low strain rates during collagen crosslinking.^193^ Fibers can also be aligned after crosslinking by taking advantage of cell contractility. As cells bind to the collagen fibers surrounding them, they generate traction forces. In cylindrical free-floating gels, the gel is compacted isotropically. However, modifying the aspect ratio and/or boundary conditions^194–196^ results in preferential fiber alignment.

Non-fibrous anisotropic hydrogels can be created by including rod-shaped microgels in a surrounding hydrogel. Microgels containing magnetic particles embedded in a PEG, fibrin, or collagen matrix have been successfully oriented using a magnetic field. The effect on cell growth and orientation depends on the differences in mechanical properties between the microgel and the embedding gel, the density of microgels, their dimensions, and their interactions with the embedding gels.^197–199^ These approaches illustrate how microscale anisotropy within hydrogels can influence cell behavior.

Similarly, hydrogels with pre-aligned fibers have deepened our understanding of anisotropy on cell behavior (Fig. 2B). They have notably provided insights into cell migration in anisotropic environments, which is a hallmark of the tumor environment.^116,191,192^ Aligned collagen matrices promote cell protrusions along the fibers, accelerating and directing cell spreading and migration in the direction of the fibers. This behavior depends on focal adhesion kinase localizations and Rac1 activity, as their inhibition eliminated protrusion anisotropy in the aligned matrices.^191^

Anisotropy within hydrogels not only influences cell migration but also mediates the organization of the surrounding matrix. Cell contractile forces cause a denser matrix immediately surrounding the cells^151^ and stiffen the local collagen fiber network at the leading edge.^200^ Cells can also align the surrounding fibers by exerting traction forces along their long axis.^194,201^ This tension-driven collagen fiber alignment enhances the effective transmission of cellular forces, increases cellular stiffness, and drives further matrix stiffening.^202,203^ Importantly, cell elongation is determined more by the organization of surrounding collagen fibrils than by the overall stiffness of the matrix,^194^ highlighting the intricate relationship between matrix structure and cellular mechanics.

### 3.3. Mechanical properties

**Fig. 3:**
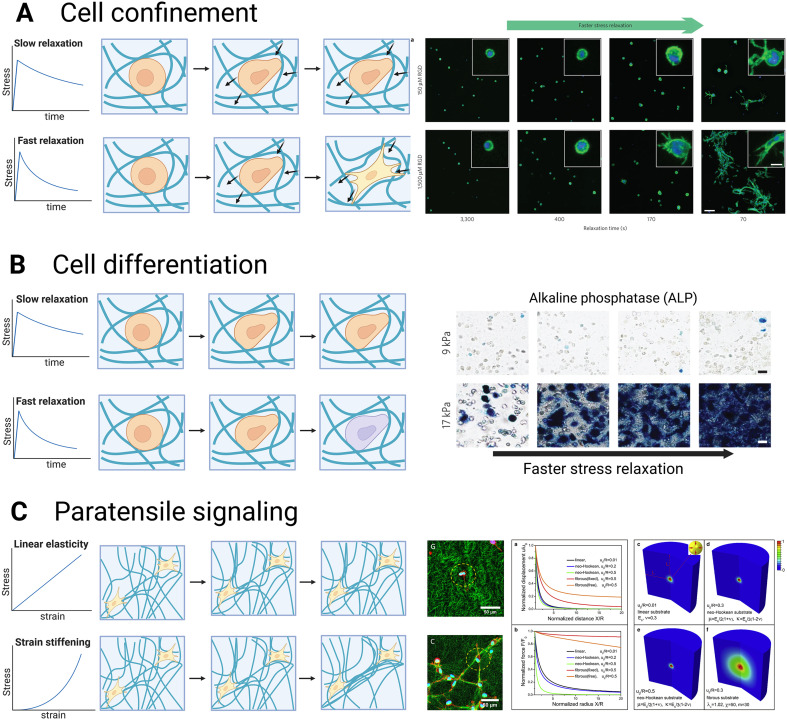
Influence of mechanics on cell behavior. (A) When the polymer network embedding the cells cannot be deformed by the cells, cells are confined and cannot spread or migrate. Fibroblasts in elastic alginate remain round while those in viscoelastic alginate can spread (reproduced from ref. 133 with permission from Nature Publishing Group, copyright 2016). (B) Viscoelasticity can influence cell differentiation. Shorter relaxation times increase stem cell differentiation into osteoblasts, as visualized by the increased ALP staining (reproduced from ref. 133 with permission from Nature Publishing Group, copyright 2016). (C) Paratensile signaling is a communication mode in which matrix fibers transmit mechanical strain between cells. This results in collagen alignment between neighbor cells (reproduced from ref. 205 with permission from Elsevier, copyright 2022). Nonlinear elasticity increases this effect as strain stiffening extends the strain field generated by cell contractility.^203^

In this section, we will focus on the methods to tune the mechanical properties of hydrogels and introduce their effect on cell behavior (Fig. 3). For a detailed review of how cells sense the mechanical properties, the mechanism of cellular mechanotransduction, and the key effectors and signaling pathways included, readers are referred to Saraswathibhatla *et al*.^26^ and Di *et al*.^204^

#### 3.3.1. Stiffness

The stiffness of natural ECM varies widely across soft tissues and physiological states, ranging from tens to several thousands Pa. Hydrogels designed to mimic the ECM must be tunable within this range. Natural polymer hydrogels, such as those formed from collagen, are typically soft (around 0–250 Pa).^206^ Common strategies to increase hydrogel stiffness include raising polymer concentration, molecular weight, or crosslinker density. These factors are governed by scaling laws that describe the relationship between stiffness and variables like polymer concentration, crosslinker density, gelling temperature, and intrinsic pore size. Hydrogels made from flexible synthetic polymers,^207,208^ and semi-flexible biopolymers with thermal^209,210^ or athermal^211–213^ networks follow distinct scaling laws, summarized comprehensively by the review of Picu.^214^ However, increasing polymer concentration also decreases pore size and, for natural ECM-derived polymers, raises ligand density, introducing additional variables. To tune stiffness while maintaining control over other properties, factors such as crosslinking temperature and pH can be adjusted to regulate pore size and bundle thickness, as detailed in Table 4.

As introduced in section 3.2.1, increasing the gelation temperature of collagen hydrogel leads to decreased pore size and fiber diameter.^179,215^ Control of both temperature and polymer concentration can modify collagen hydrogel stiffness without altering the microstructure. For instance, Yang *et al*.^148^ maintained similar pore sizes (5–7 μm) while increasing collagen I concentration from 1 to 4 mg mL^−1^, leading to a rise in *G*′ from 2 Pa to 110 Pa by adjusting gelation temperature (22 °C to 37 °C). Similarly, Seo *et al*.^161^ showed that gradually increasing gelation temperature from 4 °C to 37 °C for 6 mg mL^−1^ collagen increased pore size and fibril diameter by 20–30% while it boosted stiffness sevenfold. Changes in pH also affect collagen hydrogels: higher pH increases fibril length, decreases fibril diameter, and raises stiffness.^216^ For example, Sun *et al*.^151^ demonstrated that *G*′ rose from 5.7 Pa to 60.0 Pa as pore size decreased from 4.9 to 1.7 μm at constant collagen concentration when pH increased from 6.0 to 9.0.

#### 3.3.2. Viscoelasticity

The relaxation speed of the hydrogels (*i.e.*, their viscoelastic behavior), can be tuned independently from their initial stiffness to reproduce the range of viscoelastic behavior observed in native tissues. We present here the main methods available; for a more detailed review of various crosslinking methods and their use for constructing viscoelastic hydrogels, readers are referred to Lou and Mooney^217^ and Y. Ma *et al*.^21^

Most methods to tune the viscoelasticity focus on the polymer phase of the material by changing either the molecular weight of the polymer or the nature of the crosslinks. Decreasing the molecular weight decreases the half-stress relaxation time (*τ*_1/2_), meaning that the viscous behavior of the material increases.^88,218^ This method has been notably applied with alginate^133,219^ and HA.^220^ For example, when the average molecular weight of alginate decreased from 280 kDa to 35 kDa, the *τ*_1/2_ decreased fourteen-fold.^219^ In a study on HA-collagen interpenetrating networks, reducing the average molecular weight of HA from 120 kDa to 20 kDa resulted in a thirty-seven-fold decrease in *τ*_1/2_, whereas the molecular weight of pure HA hydrogels showed minimal impact on *τ*_1/2_ under the same conditions.^220^ Another option is to modify the type of crosslinks. Ideal static covalently crosslinked polymer networks result in an elastic material that does not exhibit viscoelasticity.^221^ In contrast, non-ideal incomplete covalent crosslinking polymers and loose ends exhibit energy dissipation, thus viscoelasticity.^88,222^ Alternatively, weak crosslinks (noncovalent physical crosslinks or dynamic covalent crosslinks) can stabilize the polymer network, giving rise to viscoelastic materials.^21,218^ For instance, a covalently crosslinked alginate network exhibits an elastic behavior, while ionically crosslinked alginate is viscoelastic.^222,223^ The introduction of reversible (dynamic) covalent crosslinking can also generate mechanically stable hydrogels with viscoelastic behavior.^224^ This type of crosslinking has been used successfully with chitosan,^225^ HA,^220^ PEG,^226–229^ and alginate.^230^ Additionally, it is possible to covalently couple different materials, such as using PEG spacers with alginate chains to decrease the *τ*_1/2_ of the alginate hydrogel, independently of the initial elastic modulus.^133,219^

Although most methods to tune viscoelasticity focus on the solid phase polymers, it is possible to tune the viscoelasticity by modifying the viscosity of the aqueous phase. Adding dextran, a homo-polysaccharide of glucose, to the aqueous phase of agarose or polyacrylamide reduces the relaxation time (*τ*) and the instantaneous elastic modulus, which represents the initial elastic response. However, it maintains similar equilibrium moduli, which represent the static response after viscoelastic relaxation during compression tests.^231,232^ This is caused by the interaction between dextran and the hydrogen bonding between water and the polymers. As less water is available to bind to the polymer chains, more water can easily flow between the polymer chains, thereby reducing the relaxation time and instantaneous modulus but maintaining the equilibrium modulus.^231^

Tuning the relaxation behavior of the hydrogels independently from the initial stiffness has unraveled the effect of each parameter on cell behavior. Decreasing the relaxation time (faster relaxation hydrogels) while maintaining the initial elastic modulus enhanced cell spreading (Fig. 3A)^133,233,234^ and osteogenic differentiation (Fig. 3B) of mesenchymal stromal cells.^133,234^ This effect was mediated through integrins and actomyosin contractility, but not through traditional focal adhesions.^133^

Viscoelasticity impacts individual cells, but also organoid development and differentiation. Hydrogels with different viscoelastic properties influence the growth, fusion, and matrix secretion of organoids from various tissues such as cartilage,^235^ kidney,^236^ intestine, and breast epithelium.^237^ Faster relaxation rates seem to improve cartilage and intestine organoid growth and differentiation, but more studies are required to draw general conclusions on the effect of viscoelasticity on organoids.

#### 3.3.3. Linearity and nonlinearity

Native fibrous tissues usually display a nonlinear mechanical behavior. Fibrous hydrogels such as collagen and fibrin can reproduce this behavior *in vitro*.^96^ In collagen hydrogels, the critical strain (*γ*_c_), marking the transition from the linear to the strain-stiffening regime, increases as the polymerization temperature rises from 26 to 37 °C, indicating enhanced non-linearity with temperature.^179^ This can be explained by decreased connectivity among fibers, from a majority of nodes with four branches to nodes with three branches.^179^ Similarly, decreasing the concentration of collagen increases the strain-stiffening behavior.^238^ Interestingly, the opposite behavior has been observed for fibrin hydrogels: the strain-stiffening behavior increases with fibrin concentration.^239^

Strain-stiffening properties are seldom observed in synthetic hydrogels.^96^ One of the few strain-stiffening synthetic polymers is PIC, which shows a low critical stress *σ*_c_ of tens of Pa and a stiffening index *m* of about 3/2, similar to biopolymer hydrogels.^240,241^ Keeping PIC concentration constant, increasing polymer chain contour length *L* will increase the plateau modulus *G*_0_ and critical stress *σ*_c_ with *G*_0_∝*L^2^* and *σ*_c_∝*L*,^242^ while decreasing *m*,^241^ resulting in stiffer and less stress-sensitive gels. Addition of an increased amount of peptide sequence GRGDS to PIC polymers will decrease *G*_0_ and *σ*_c_ exponentially, thus the strain-stiffening responsiveness is higher.^243^

Nonlinear hydrogels have improved our understanding of cell–cell mechanical communication. Nonlinear elasticity increases cell–cell mechanical communication *via* paratensile signaling, a communication mode in which mechanical strain is transmitted between cells *via* matrix fibers^35^ (Fig. 3C). This effect is enhanced by strain stiffening as it extends the strain field generated by cell contractility^244^ and thus promotes long-range mechanical signaling.^202,245^ The importance of strain-stiffening for mechanical communication depends on the cell type. For neurons, the strain-stiffening is not obvious considering the lower level of stress that neurons can exert on the surrounding matrix. For cells that can induce rather large stresses through contractility such as fibroblasts,^246^ hMSCs,^245,247^ or breast cancer cells,^202,248^ the effects of strain-stiffening become obvious. This phenomenon is critical in the context of fibrosis. Myofibroblasts embedded in a collagen gel can generate force, which is then transmitted through fibrous collagen, and activate quiescent fibroblasts embedded in the same gel, thus propagating fibrosis.^249^

### 3.4. Hydrogels as a dynamic environment

As discussed in section 2.4, the mechanical properties and morphology or structure of the extracellular matrix (ECM) evolve, and several studies have aimed to replicate these temporal changes, usually using stimuli-responsive hydrogels. The stiffness, swelling ratio, and structure of these hydrogels can respond to various stimuli such as light, temperature, pH, magnetic field, humidity, biomolecules, and cell force.^250,251^ Here, representative studies on stimuli-responsive hydrogels compatible with embedded cells are introduced and categorized based on the applied stimuli.

Photopolymerization is a common technique for manufacturing photoresponsive hydrogels.^252^ Photopolymerization can induce a second crosslinking step to increase stiffness^135,253^ or degrade the gel to reduce stiffness.^254,255^ Recently, PEG–alginate hydrogels have been used to tune the relaxation rate of the gels over time *via* photopolymerization of the PEG network.^256^ Incorporation of PEG molecules in the alginate network decreased the *τ*_1/2_. Though the *τ*_1/2_ was still higher than the characteristic *τ*_1/2_ of most biological tissues (∼10 s), the authors produced gels with relaxation times varying from 80 to 800 seconds. As this method is compatible with cell encapsulation, the relaxation time of the gels can be tuned during cell culture. Similarly, norbornene–HA hydrogels allow spatiotemporal control over stiffness and relaxation rate upon photocrosslinking.^257^ Despite these promising results, it is important to note that photocrosslinking can be toxic for cells because of the photoinitiator used and the type of light required.^258^

Temperature- and pH-responsive hydrogels have been widely explored for regenerative medicine applications. For example, injectable hydrogels designed to gelate at physiological temperatures (20–37 °C)^259,260^ or specific pH conditions^261,262^ have been developed to encapsulate cells for *in vivo* treatments, such as heart tissue regeneration or stem cell delivery. While these applications are promising, they fall outside the scope of this review, which focuses on hydrogels as ECM mimics for studying cell behavior *in vitro*.

Other environmental stimuli include magnetic fields and hydration. Magnetic fields can tune the stiffness and induce anisotropy, guiding cell alignment,^263,264^ while 4D bioprinting makes use of the degree of hydration to induce bulk shape.^265^

Hydrogels can also respond to cellular stimuli, such as MMP secretion^266^ or traction forces. For example, predetermined patterns of mesenchymal cells can dynamically fold collagen substrates into certain desired patterns, such as hollow tube structures. The folded structure generates vessels when the substrate is seeded with HUVEC.^267^

Recently, Major *et al*. have implemented a hydrogel showing a progressively stiffening of compressive modulus for about 10 to 15 kPa without requiring external stimuli during the 3 week culturing time. The hydrogel is composed of adipose-derived ECM and silk fibroin, which can be initially photo-crosslinked by visible light and added photoinitiators. The stiffening is due to the spontaneous formation of β-sheet secondary structures through hydrogen bonding in silk fibroin after crosslinking and during the culture time.^268^

### 3.5. Combination of materials

Though we have discussed hydrogels composed of single polymer types, it is sometimes advantageous to combine different types of polymers, to leverage both properties or to introduce heterogeneities in the hydrogels. Interpenetrating polymer networks (IPNs) are an example of hydrogels formed by combining at least two crosslinked polymer networks that are entangled but not chemically linked.^269^ IPNs can be made from ECM-derived polymers, such as collagen and fibrin,^270^ or collagen and Matrigel.^271^ In these cases, the architecture of the collagen network is altered in different ways: incorporating Matrigel decreases the collagen fiber length, whereas incorporating fibrin results in longer fibers. Changes in mechanical properties accompany these architectural changes; Matrigel reduces the nonlinear behavior of collagen, while fibrin decreases the viscoelastic behavior of the collagen network.

IPNs can also combine ECM-derived polymers with polymers lacking cell adhesion sites, enabling tuning the mechanical properties by modifying nonfibrous components without changing protein concentration or the structure of fibrous protein components. However, the combination of different polymers changes the bulk behavior of the material. A well-known type of IPN is alginate–collagen IPN, in which collagen fibrils network intercalate with alginate mesh. Ionic crosslinking of alginate keeps the IPN fibrous architecture and mesh size unchanged. Low concentration (<2 mM) or no Ca^2+^ for crosslinking alginate leads to IPN storage modulus close to pure collagen hydrogel of the same concentration, and increasing Ca^2+^ concentration from 2.5 mM to 10–15 mM leads to stiffness increase from tens of Pa to about 1 kPa.^171,272^ Another example is IPN gels made of collagen and GAG, notably hyaluronic acid. Although HA has multiple cell receptors, a study showed that its effects are similar to that of inert alginate when combined with collagen, proving that the observed effects were mechanical rather than biochemical.^273^ Compared with a pure collagen gel of the same concentration, adding HA to the collagen network showed a two- to four-fold increase in *E*^153,156,157^ or a fifteen-fold increase in *G*′,^159^ while maintaining approximately the same pore diameter and fiber diameter. However, adding other GAG (*eg*, chondroitin sulfate, dermatan sulfate) to collagen to form hydrogel did not influence stiffness.^156^ Incorporating HA in collagen gels reduced cell-traction forces and cardiomyocytes alignment.^273^ Additionally, HA in collagen gels also reduces plastic deformation.^274^

The combination of two distinct polymer networks, where the fibrous component is not derived from ECM proteins, can also create IPNs. For instance, Chen *et al*.^162^ developed a fibrous hydrogel using aldehyde-modified cellulose nanocrystals (a-CNCs) as the structural component and incorporated gelatin methacryloyl (GelMA) for intrafibrillar photocrosslinking without affecting interfibrillar crosslinking among a-CNCs. By increasing the degree of GelMA crosslinking—either through higher methacryloyl group content or prolonged photoirradiation—*G*′ was enhanced from several kilopascals to over 10 kPa, while maintaining consistent a-CNC and GelMA concentrations, as well as pore and fibril diameters. This demonstrates how tuning intrafibrillar crosslinking can significantly modulate hydrogel stiffness without altering other structural parameters.

While IPNs generally result in homogeneous hydrogels at the cell scale, heterogeneous hydrogels can be created using large inclusions of sacrificial materials to form large void structures. These templating methods involve using a pre-existing structure or template to guide the creation of desired porosity in the final hydrogel material.^142^ One common technique involves incorporating solid particles (porogens) into the hydrogel precursor, which are then removed after crosslinking to create larger pore sizes, ranging from tens to several hundreds of micrometers.^143,275,276^ Gelatin is a good porogen as it is biocompatible, dissolves at 37 °C, and is soluble in water. Incorporating gelatin beads allows for tuning pore size independently of porosity. This method has been successfully used with PEG,^277^ alginate,^278^ and various photocrosslinkable bioinks.^276^ This principle can be extended to any cytocompatible sacrificial material. For example, lyase-digestible alginate and alginate resistant to lyase digestion have been patterned to create microchannels after digestion.^279^

The inclusion of larger pores by using sacrificial material allows us to uncouple the effects of porosity from pore size on cell behavior. Porosity (26% *vs.* 65%) influenced cell growth in PEG gels with pores created by leaching gelatin, while pore size (122–233 μm) had no effect. Similarly, porosity, but not pore size, influenced cell spreading.^277^ Osteoblast-like cells cultured in bioprinted constructs also showed enhanced proliferation with 40% porosity and pore sizes of the cell scale (40 μm). Furthermore, matrix mineralization appeared more uniform in the porous constructs than in the bulk hydrogels after 14 days.^276^ Larger porosities forming channels inside the hydrogel enable the connection to a perfusion system, which supports cell proliferation and tissue formation.^279^ Thus, porogens can be used to enhance nutrient diffusion and waste removal to compensate for the reduced diffusion in the core of hydrogels.

### 3.6. Interplay between properties

**Fig. 4:**
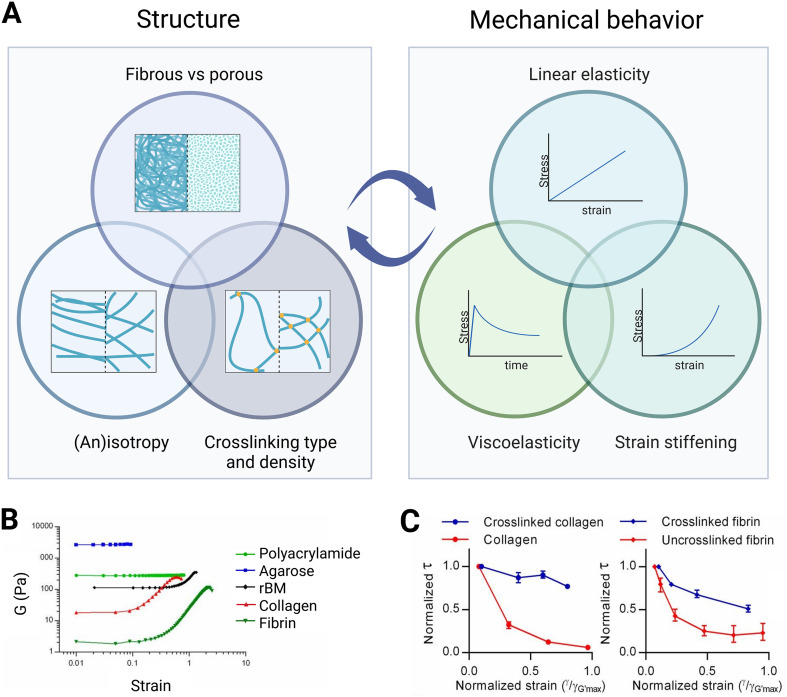
Interplay between structural and mechanical properties. (A) Structural properties and mechanical behavior of hydrogels are interdependent and interrelated with no simple one-to-one correspondence. For example, increasing the crosslinkers will increase stiffness but also reduce the intrinsic porosity of the polymer network. The presence of fibers confers a nonlinear behavior, while porous hydrogels have a more linear behavior. Nonlinearities and crosslinking types both influence the relaxation behavior. Fiber anisotropy leads to mechanical anisotropy with higher stiffness in certain directions. (B) Collagen and fibrin exhibit strain-stiffening, but not agarose or polyacrylamide. (C) Strain-stiffening and viscoelasticity in natural hydrogels influence each other. Crosslinking partially cancels this effect. (B) and (C) are reproduced from ref. 94 with permission from National Academy of Sciences, copyright 2016.

Although we discussed structural and mechanical properties separately, they are interrelated and influence each other significantly (Fig. 4A). For natural polymers derived from the ECM, increasing polymer concentration raises the elastic modulus, while simultaneously reducing pore size and increasing the density of ligands to which cells can bind,^280^ thus introducing additional variables (Table 4). In synthetic polymers, ligand density can be adjusted independently of polymer density,^133,134^ yet polymer density still correlates with pore size.

Both intrinsic pore size (or mesh size) and mechanical properties influence cell migration. If the mesh size is larger than the cell nucleus, cells can move freely. If the mesh size is smaller, cells must either degrade or deform the matrix. Cells can migrate through a matrix that is plastic enough and of nanometer-scale intrinsic pore size without using proteases, by using protrusions to open up micrometer-scale channels for migration.^281^ If the gels are elastic and too stiff for the cells to deform, this creates a mechanical confinement for the cells, which negatively impacts various biological processes.^230,282,283^ Viscoelasticity also enables cell spreading at constant stiffness and pore size,^133,284^ as previously mentioned in section 3.3.2. However, increasing mesh size decreases stiffness,^54,207–214^ making it difficult to separate stiffness from mesh size. Thus, while mesh size influences overall stiffness, factors such as stiffness, viscoelasticity, and plasticity also affect how intrinsic pore size impacts cells. It is worth noting that while this is true for the intrinsic porosity (mesh size), the impact of larger pores created by including porogens is different: in this context, porosity but not pore size influences the bulk stiffness. Besides, the local stiffness (cell scale) remains unchanged.^277^

In addition to pore size, structural anisotropy also impacts mechanical properties as structural anisotropy leads to mechanical anisotropy. Consequently, the effects of these two properties are difficult to decouple. For collagen hydrogel with the same concentration, anisotropically oriented collagen fibers enhance the hydrogel elastic modulus along the fiber orientation compared to hydrogels with isotropically oriented fibers.^285^ The anisotropic distribution and stiffness of matrix fibers stabilize cell protrusions in the direction of the alignment and promote cell migration,^280^ as well as fibroblast to myofibroblasts transition.^286^ However, increased elastic modulus alone through crosslinkers does not promote cell elongation.^280^

Besides the reciprocal influence between mechanical and structural properties, mechanical properties can also interact and influence each other. For instance, Nam *et al*.^94^ demonstrated the interplay between relaxation and strain-stiffening in fibrous hydrogels. Fibrin and collagen exhibit both strain-stiffening (Fig. 4B) and viscoelasticity (Fig. 4C). The higher the initial strain, the faster the stress relaxation, leading to a rapid decrease in the strain-stiffening effect over time. Numerical simulations suggested that force-dependent unbinding followed by rebinding of fibers can explain stress-enhanced stress relaxation in collagen networks. Covalent crosslinking reduces this effect. Thus, increasing collagen stiffness through transglutaminase or glutaraldehyde crosslinking also alters the material's viscoelastic and nonlinear response. With the aid of magnetic microrheometry and a probabilistic modeling approach to enhance the analysis of the sparse probe-generated data, Arasalo *et al*.^246^ recently reported an enhanced measurement and mapping of the spatial heterogeneity of viscoelasticity and stiffness in a 3D collagen hydrogel embedded with cancer-related fibroblasts. Their study observed collagen stiffening and bundle formation in the collagen matrix in the vicinity of cells, and a decrease in the collagen phase angle, indicating a more elastic behavior, which relates to the stiffened collagen bundle formation by cell force.

It is important to note that while stiffness is commonly reported, other mechanical properties (such as viscoelasticity and nonlinearity) and structural properties (such as pore size and the presence of fibers) are rarely mentioned or discussed. This may explain why some studies report conflicting results regarding the effect of stiffness on cell behavior. For example, Hadjipanayi *et al*.^287^ observed 3.5 times more proliferation of fibroblasts over 2 days in collagen gels at 143 kPa compared to gels at 42 kPa. Conversely, Shie *et al*.^288^ found twice as much proliferation of fibroblasts in 50 kPa GelMA gels compared to 139 kPa gels. The differences in the viscoelastic behavior of these materials may explain such opposite observations; collagen exhibits viscoelastic behavior, whereas GelMA is more elastic. Chaudhuri *et al*.^133^ proposed a mechanism to explain enhanced cell spreading and proliferation in viscoelastic matrices. A cell embedded within a three-dimensional matrix initially imparts strains on the matrix, leading to forces and stresses that resist this strain. In purely elastic matrices, these forces remain constant, preventing any remodeling of the matrix microenvironment. In viscoelastic matrices, however, forces within the matrix can gradually dissipate over time. The extent of mechanical remodeling depends on the rate of stress relaxation. In matrices with rapid relaxation, this process facilitates adhesion-ligand clustering, changes in cell shape, and proliferation.

Differences in ligand densities can also explain discrepancies between studies. Increasing collagen density to raise the elastic modulus also increases ligand density, while increasing the crosslinking of GelMA does not affect it. Moreover, gelatin, unlike collagen, has lost its ability to form fibers, which also impacts cell behavior.^289^ Thus, several factors can explain the contrasting cell growth rates observed in these studies.

## 4. Conclusion and outlook

The extracellular matrix (ECM) surrounding cells is a complex biochemical and mechanical environment that undergoes constant remodeling in response to mechanical loading, injury, or disease. These dynamic interactions significantly influence cell behavior and play a crucial role in maintaining healthy tissue. To better understand how these cues affect cellular functions, it is essential to develop *in vitro* models that can decouple these factors. Hydrogels emerged as promising candidates for this purpose, as they can mimic the biphasic behavior of the ECM and offer tunable properties. Both natural and synthetic polymers, either alone or in combination, can be employed to replicate the biochemical and mechanical characteristics of the ECM. Thanks to these hydrogels, we have learned how cells respond to the structural (pore size and anisotropy) and mechanical properties (stiffness, viscoelasticity and nonlinearity) of the ECM. However, given the interconnected nature of these factors and the difficulty of varying a single property without inadvertently altering others, it remains challenging to isolate the effect of single ECM parameters on cell behavior. This highlights the need to go beyond the simple reporting of stiffness: complete mechanical as well as structural characterizations should be provided.

Besides, the next generation of hydrogels should involve strategic combinations of multiple polymers to better replicate physiological conditions and achieve more precise control over these variables. Because of the aforementioned intertwining of structural and mechanical factors, designing such hydrogels with desired properties typically requires laborious experiments with trial and error. The recent rapid development of artificial intelligence (AI) and its subfield machine learning (ML) has already allowed more efficient optimization of parameters for 3D and 4D printing of hydrogel.^290–292^ Such approaches can also be further developed as promising tools to advance the design of functional hydrogels,^293–295^ possibly *via* the strategy of simultaneously combining hydrogel compositions and structural properties to predict their influence on the mechanical properties of multi-component hydrogels, in combination with cellular mechanobiology to address their complex impact on “what cells see” and “what cells feel”.

## Author contributions

J. S.: conceptualization, writing – original draft, writing – review & editing, visualization. S. P.: conceptualization, writing – original draft, writing – review & editing, visualization. N. A. K.: writing – review & editing, supervision, funding acquisition.

## Conflicts of interest

There are no conflicts to declare.

## Data Availability

No primary research results, software or code have been included and no new data were generated or analysed as part of this review.
